# The harm reduction shift: A review of India's NACP strategies for PWID

**DOI:** 10.6026/973206300222487

**Published:** 2026-04-30

**Authors:** Kapil Prajapat, Fathima Hassan, Lulu Sirin Chamayi, Rislaj Hameed Koduvayalil, Ankit Das, Darshit Samatbhai Jinjala, Vedant Mukul Joshi

**Affiliations:** 1 Department of Community & Family Medicine, All India Institute of Medical Sciences, Bhopal, Madhya Pradesh, India; 2 MBBS, Azeezia Institute of Medical Sciences & Research, Kollam, Kerala, India; 3 Department of General Medicine, All India Institute of Medical Sciences, Bhopal, Madhya Pradesh, India; 4 MBBS, All India Institute of Medical Sciences, Bhopal, Madhya Pradesh, India

**Keywords:** Human Immunodeficiency Virus (HIV), National AIDS Control Programme (NACP), People Who Inject Drugs (PWID)

## Abstract

India's HIV epidemic is highly concentrated among People Who Inject Drugs (PWID) and who exhibits the highest prevalence among key populations despite decades of intervention. The Review highlights a policy shift from awareness-based campaigns in NACP-I to Targeted Interventions in NACP-II, the three-tier harm-reduction model including Opioid Substitution Therapy (OST) under NACP-III and differentiated care approaches, such as satellite centres and prison interventions, in NACP-IV and V. By 2023-24, programme expansion achieved 90% intervention coverage, 91.3% reported use of sterile injecting equipment and OST enrolment of over 54,000 individuals. However, HIV prevalence among PWIDs rose from 6.26% in 2016 to 9.03% in 2021. This shows indicating that service expansion has not fully translated into epidemic control.

## Background:

HIV/AIDS remains a major global public health challenge. In 2024, an estimated 40 million people were living with HIV worldwide, with over 1.3 million new infections reported that year [1]. In South Asia, India carries the largest share of the HIV burden, accounting for nearly 80% of all people living with HIV in the region [2]. In India, an estimated 2.3-3.1 million people are living with HIV, making it the third largest epidemic globally [3]. Although national prevalence remains below 1%, the epidemic is highly concentrated among key populations, especially People Who Inject Drugs (PWID), who exhibit markedly higher HIV prevalence than other high-risk groups [4]. Unsafe injecting practices, frequent needle sharing, incarceration and vulnerabilities such as homelessness, poverty and stigma place PWIDs at the centre of a persistent and geographically expanding concentrated epidemic [5]. Efforts to end the HIV/AIDS epidemic are now closely aligned with global development priorities, particularly the UN Sustainable Development Goals (SDGs), which call for ending AIDS and other major epidemics by 2030. This mandate has shaped international strategies focused on reducing new infections and AIDS-related deaths through high-impact prevention, timely diagnosis and treatment and improved access to quality health services for people living with HIV [6]. In India, the National AIDS Control Programme (NACP) has evolved over three decades from awareness-driven initiatives in NACP I to structured Targeted Interventions, comprehensive harm-reduction packages, expanded Opioid Substitution Therapy (OST) and Test-and-Treat strategies under NACP IV and V. Despite these advances, PWIDs continue to exhibit the highest HIV prevalence among key populations, hindered by structural barriers such as healthcare stigma, incomplete Needle Syringe Exchange Programme (NSEP) coverage, inconsistent OST availability, challenges in population size estimation and weak linkage from HIV testing to ART [7]. Therefore, it is of interest to describe the evolution of India's national HIV policy for PWIDs across NACP phases, highlighting key achievements, strategic gaps, implementation barriers and future priorities to strengthen the harm-reduction ecosystem and advance progress toward the UNAIDS 95-95-95 targets and HIV elimination by 2030.

## Evolution of strategies for PWID from NACP I TO V:

## Early recognition of PWID under NACP (1992-1999):

This phase focused on awareness generation, establishing HIV surveillance and ensuring access to safe blood and preventive services for high-risk groups [8]. Early surveillance revealed unacceptably high HIV prevalence among PWIDs [9].

## Formal adoption of targeted interventions and harm reduction in NACP-II (1999-2006):

NACP-II marked a shift from broad awareness to targeted interventions. Recognizing PWIDs as a key high-risk group, India formally adopted a harm-reduction policy in 2002. Core services included behaviour change communication, counseling, abscess management, NSEP, STI treatment and referral for HIV testing and care. These interventions were implemented through NGO-led TI projects in high-prevalence states [10].

## Scaling of targeted interventions and introduction of structured harm reduction (2006-2012, NACP-III):

This phase introduced a comprehensive harm-reduction package, including abscess management, STI services, and HIV testing and peer outreach. A key feature was the structured three-tier harm-reduction model. Tier 1 focused on outreach and needle-syringe exchange; Tier 2 introduced OST; and Tier 3 strengthened linkages to services such as DOTS, ICTC, ART, reproductive health care and drug-dependence treatment. Unlike NACP-II, which emphasised only Tiers 1 and 3, NACP-III expanded and institutionalized OST as a central component [11]. HIV sentinel surveillance expanded under NACP-III, reaching 63 sites to monitor HIV prevalence among PWIDs. A major focus was scaling up TI services, resulting in a threefold increase in PWID-exclusive TIs to 261 centres, with most growth occurring outside the Northeast. NACP-III also introduced OST for the first time, establishing 52 OST TI centres that served 4,810 clients, along with five pilot sites in Punjab, which reached approximately 500 clients. Despite this expansion, National sentinel surveillance showed persistently high HIV prevalence among PWIDs (9.2%), the highest among all key populations, with newer epidemic areas such as Punjab, Odisha and Bihar also reporting elevated rates. The operational definition of PWIDs, limited to those injecting in the past three months, excluded infrequent injectors and non-injecting users, leaving many at-risk individuals outside programme coverage. Although one-time TI coverage reached about 80%, regular service uptake and consistent sterile injecting practices remained low. Needle-syringe distribution improved but stayed suboptimal at <100 needles per injector per year, well below recommended levels. Programme quality was further affected by inadequate staffing structures, stagnant salaries, limited budget flexibility, delayed OST scale-up and poor commodity rationalization. Against a target of 20% OST coverage, only 2-3% of PWIDs were enrolled, with major disparities between NGO and government centres. Additional challenges included limited options for OST medication, the absence of uniform accreditation systems and insufficient capacity-building among service providers, SACS, STRCs and monitoring teams. TI-based delivery also failed to address broader needs such as overdose prevention, hepatitis care, nutrition, shelter and affordable detoxification services. Stigma related to drug use and HIV continued to deter service uptake, while weak inter-ministerial coordination constrained support systems. ART registration and adherence among PWIDs remained very low and gender-specific needs, particularly of female PWIDs and partners of male PWIDs, were poorly addressed, signaling the need for more gender-responsive interventions. To strengthen quality, extensive capacity-building initiatives produced standardised resource materials, including waste-management guidelines; harm-reduction and counseling modules, SOPs, OST practice guidelines and manuals for working with partners of PWIDs [12].

## Under NACP-IV and extension (2012-2021):

Under NACP Phase IV and its extension, harm-reduction strategies for PWIDs evolved into a more comprehensive "prevention-to-care" model. OST became a central component, shifting services from basic needle exchange to a musicalized approach using buprenorphine and methadone. To improve access and retention, a differentiated prevention model was introduced in 2017, including Satellite OST centres to decongest main sites and reach remote areas. In 2019, "low threshold" approaches were added to further reduce entry barriers. The Programme also strengthened the linkage of PWIDs to ART refills directly through TI centres, improving continuity between prevention and HIV care. By 2019-20, NACP-IV employed multiple service-delivery models to reach PWIDs, operating 42 NGO-run OST centres, 182 collaborative centres in government hospitals and 51 satellite centres. HIV prevention, treatment and OST services were also expanded into prisons, covering 971 of 1,363 facilities. Recognizing gender-specific vulnerabilities, states like Manipur introduced the FPWID Intervention Model, offering core services plus sexual and reproductive health care, gender-focused mental health support and social-protection linkages. The Link Worker Scheme was revamped in 2016 to better connect rural HRGs with formal health services. Despite achieving the coverage target of 1.65 lakh PWIDs and improving HIV testing uptake from 62% to 72%, epidemiological trends remained concerning. HIV positivity rose from 0.85% in 2013-14 to 1.11% in 2019-20 and PWIDs continued to have the highest HIV prevalence nationally (6.26%), even as prevalence declined among FSWs and MSM. Service utilisation indicators also dropped, including condom distribution and STI detection, pointing to gaps in comprehensive screening. PWID interventions were the most resource-intensive, with significantly higher per-person costs than other HRG programmes, although OST remained cost-efficient. Qualitative findings highlighted persistent gaps, including the absence of integrated Hepatitis C care, challenges in obtaining government IDs to reduce harassment and service disruptions during the COVID-19 pandemic [13].

## NACP V (2021-2026):

Under NACP Phase V (2021-2026), PWIDs remain a high-priority group, with HIV prevalence estimated to be 7-28 times higher than in the general population. The strategy acknowledges regional heterogeneity, noting that while sexual transmission dominates nationally, injecting drug use contributes to at least 25% of infections in states such as Manipur, Mizoram, Tripura and Punjab. To address this, Goal 1 calls for intensified expansion of comprehensive harm-reduction services, particularly the Needle-Syringe Exchange Programme (NSEP) and OST. The plan aims to increase OST centres from 232 in 2021 to 600 by 2025-26, targeting OST coverage for 0.63 lakh PWIDs. NACP-V also promotes a convergent approach by partnering with the Ministry of Social Justice and Empowerment (MoSJE) and establishing structured referral pathways with the National Viral Hepatitis Control Programme (NVHCP) to address the high burden of Hepatitis C co-infection [14]. In Figure 1 (see PDF), the HIV sero-prevalence among PWIDs in India has shown substantial fluctuation over the last two decades [15].

## Performance of PWID Interventions in India:

Table 1 (see PDF) demonstrates a consistent upward trajectory in service infrastructure and uptake between 2016 and 2024 [16, 17, 18, 19, 20-21].

## Conclusion:

Despite significant expansion of harm-reduction services and high coverage levels, the continued rise in HIV prevalence among PWIDs highlights that access alone is insufficient to control transmission. Achieving the 2030 elimination targets will require a strategic shift from scale to quality, with emphasis on differentiated care, addressing the needs of female PWIDs, integrating Hepatitis C services and strengthening the continuum from testing to sustained ART adherence. A targeted, rights-based and quality-driven approach is therefore essential to convert programme reach into meaningful epidemiological impact.
